# Blood–brain barrier permeability changes in dogs with suspected canine cognitive dysfunction using magnetic resonance imaging subtraction enhancement analysis

**DOI:** 10.3389/fvets.2025.1572286

**Published:** 2025-05-13

**Authors:** Yael Merbl, Ella Mondrus, Erez Hanael, Merav H. Shamir

**Affiliations:** ^1^Cornell Hospital for Animals, Department of Clinical Sciences, Cornell University, Ithaca, NY, United States; ^2^Veterinary Teaching Hospital, Koret School of Veterinary Medicine, Hebrew University of Jerusalem, Rehovot, Israel

**Keywords:** blood-brain barrier, subtraction enhancement, canine cognitive dysfunction, dogs, MRI

## Abstract

**Background:**

Blood–Brain Barrier (BBB) breakdown/ dysfunction (BBBD) has been recognized as a contributing factor to cognitive decline in degenerative diseases and the normal aging process in the elderly. There is a need for antemortem evaluation tools to assess the permeability of the BBB in cases of canine cognitive dysfunction (CCD), allowing for better grading of the dysfunction and monitoring of its progression.

**Hypothesis/objectives:**

This study aims to examine changes in the BBB permeability using magnetic resonance imaging (MRI) in dogs with CCD compared to a control group. We hypothesized that changes in BBB permeability would be detected and quantified using subtraction enhancement analysis (SEA).

**Animals:**

MRI scans of dogs with signs of CCD were received from the Koret Veterinary Teaching Hospital (*n* = 10, 0.35 T MRI) and WSU (*n* = 3, 1.5 T MRI) and compared to non-CCD dogs (*n* = 9 from Koret, *n* = 6 from WSU).

**Methods:**

This is a retrospective case–control study. MRI scans were analyzed using SEA to determine a threshold value of “positive-permeable” voxels, which was then used to highlight suspected areas and calculate a score for BBB dysfunction (BBBD).

**Results:**

Mean BBBD scores did not differ significantly between the study and control groups. BBBD was present in a few cases of CCD, but not in all.

**Conclusion and clinical importance:**

SEA was less effective in recognizing BBBD in dogs with CCD compared to those with other canine diseases such as neoplasia and seizures. It may be necessary to explore alternative methods to increase the sensitivity of BBBD detection for CCD, or it may that BBBD occurs only in a subpopulation of patients.

## 1 Introduction

Over the last several decades, improvements in veterinary medicine have resulted in an increase in the elderly dog population and consequently an increase in age-related medical conditions. Among these age-related conditions is a clinical manifestation known as canine cognitive dysfunction (CCD), also known as “Cognitive Dysfunction Syndrome” or canine dementia. It is a common syndrome in older dogs, affecting up to 60% (1, 2). CCD is characterized by a gradual decline in cognitive ability due to progressive neurodegenerative changes in the brain. A set of clinical signs, known by the term “DISHA,” characterizes the condition. These signs include, but are not limited to, disorientation, changes in social interaction, sleep–wake cycle, housetraining, and activity deficits (3, 4). In a similar way to humans, cognitive decline in dogs includes impairments in memory, learning skills, awareness, and perception (5, 6).

It is currently assumed that less than 2% of dogs are diagnosed (4) with CCD, which may be due to the misperception of owners attributing the signs to normal aging processes. Additionally, there are no tests or specific biomarkers for the diagnosis of CCD. Currently, the mainstay of diagnosis of CCD relies on a physical and neurological examination, elimination of other pathologies, and owners’ scoring questionnaires (7), and, in some cases, the support of an MRI showing signs consistent with brain atrophy (8–10). These signs may include widened sulci, thinned parenchyma, and ventricular dilatation (8–10) and a decreased size of the interthalamic adhesion (11). A specific and reliable diagnostic tool to differentiate between normal and pathological aging in the dogs’ population is warranted. A previously described contrast-enhanced method, allowing the quantification of the permeability of the BBB, named “subtraction enhancement analysis” (SEA), could potentially identify BBB leakage and permeability and be used as a biomarker for an early state of CCD (12). In general, subtraction imaging is a technique whereby an unenhanced T1-weighted sequence is digitally subtracted from the identical sequence performed after gadolinium administration.

Using SEA to identify and quantify BBB leakage in CCD patients may reveal associations between the occurrence and extent of BBBD, as well as clinical diagnosis of CCD, shedding more light on the pathophysiology of this syndrome.

In a previous study in dogs suffering from encephalitis, BBBD was identified in 53% when the SEA method was applied. Furthermore, applying the SEA method on MRI studies retrospectively improved the ability to identify brain abnormalities from 50 to 72% in dogs with MUO. Subtraction techniques have also been studied in humans with dementia and have been reported to have potential in increasing sensitivity to recognize abnormalities (13).

The advantage of SEA compared to other dynamic MRI methods is that it can be applied to previously acquired MRI images and does not require additional sequences other than those routinely acquired for clinical indications.

The main objective of this study was to examine whether changes in BBB permeability could be detected using SEA on MRI images from dogs with suspected CCD and whether this technique could serve as a diagnostic biomarker for CCD. We hypothesized that dogs with CCD present changes in BBB permeability compared to a control group, resulting in higher BBBD scores.

## 2 Materials and methods

### 2.1 Animals and study design

This study is a retrospective case–control study conducted at two institutions, using MRI images of dogs suspected of suffering from CCD and comparing them to those without CNS disease. Medical records were searched based on the following inclusion criteria: (1) Dogs admitted to either the Koret Veterinary Teaching Hospital (KVTH) or to Washington State University (WSU) between 2010 and 2018 with clinical signs compatible with CCD. (2) Dogs were included in the study if they had a diagnostic MRI performed and brain atrophy suspected. Dogs were excluded from the study group if other CNS pathologies were found. Sequences obtained for clinical purposes included T1-weighted image (WI), T2WI, T2W Fluid-attenuated inversion recovery (FLAIR), T2W gradient echo, T2W Short Tau Inversion Recovery (STIR) WI, and T1WI post-contrast. Additionally, diffusion-weighted imaging (DWI) and a calculated apparent diffusion coefficient (ADC) map were obtained at WSU. Of those, for this study, transverse planes of T1WI pre- and post-contrast were used for further analysis. The main method in this work is termed subtraction enhancement analysis (SEA) and described herein.

### 2.2 Subtraction enhancement analysis

For KVTH, SEA was performed using the following MRI sequences: T1-weighted spin echo (SE) in transverse plane (TE: 21 ms, TR: 642–916 ms, flip angle: 90°, slice thickness: 3.5–4.0 mm).

For WSU, SEA was performed using the following MRI sequences: T1-weighted SE in transverse plane (TE: 15 ms, TR: 674.1–800.9 ms, flip angle: 90°, slice thickness: 3.5 mm).

Dogs from KVTH were divided into two groups: Group A included 10 dogs that presented with cognitive signs such as decreased mentation and/or behavioral changes, which led to a tentative diagnosis of CCD (KVTH-CCD) with no other CNS pathological abnormalities on MRI. Group B included nine control dogs that had an MRI without any indication of CNS diseases (KVTH-control). These included dogs with old dog vestibular syndrome, otitis media, and other non-CNS conditions.

Dogs from WSU were divided into similar groups: Group A included three dogs that presented cognitive signs of CCD, such as mentation changes or behavioral changes, but had no noticeable pathological abnormalities in their MRI report other than signs of brain atrophy. Group B included six dogs who did not exhibit CNS signs and served as a control group. Clinical signs and findings of the dogs in this group included peripheral vestibular signs, limb weakness, and intervertebral disc bulging.

### 2.3 Image acquisition and analysis

MRI DICOM files were collected from both institutions.

All MRI studies from WSU were conducted on the same 1.5-T Tesla magnet (General Electric, unknown model), with an appropriate surface coil matched to the size of the patient. T1W images post-contrast were acquired after administration of 0.2 mL/kg of gadobenate dimeglumine (Bracco Diagnostics Inc., Monroe Twp., NJ) via intravenous bolus.

All MRI studies from KSVH were performed on a 0.35-Tesla magnet (Magnetom C, Siemens Healthineers, Berlin, Germany) using an appropriate surface coil matched to the size of the patient. T1W images were taken post-contrast after the administration of 0.3 mL/kg gadolinium-diethylenetriamine pentaacetate (Magnetol) via intravenous bolus.

All MRI scans used for this study were part of the clinical workup. Additional analyses were performed by the raw MRI data obtained from the radiology archive of the institutions.

The T1-weighted sequences taken before and after the injection of contrast were used for the SEA, which was performed using in-house MATLAB scripts (14).

**Figure 1:**
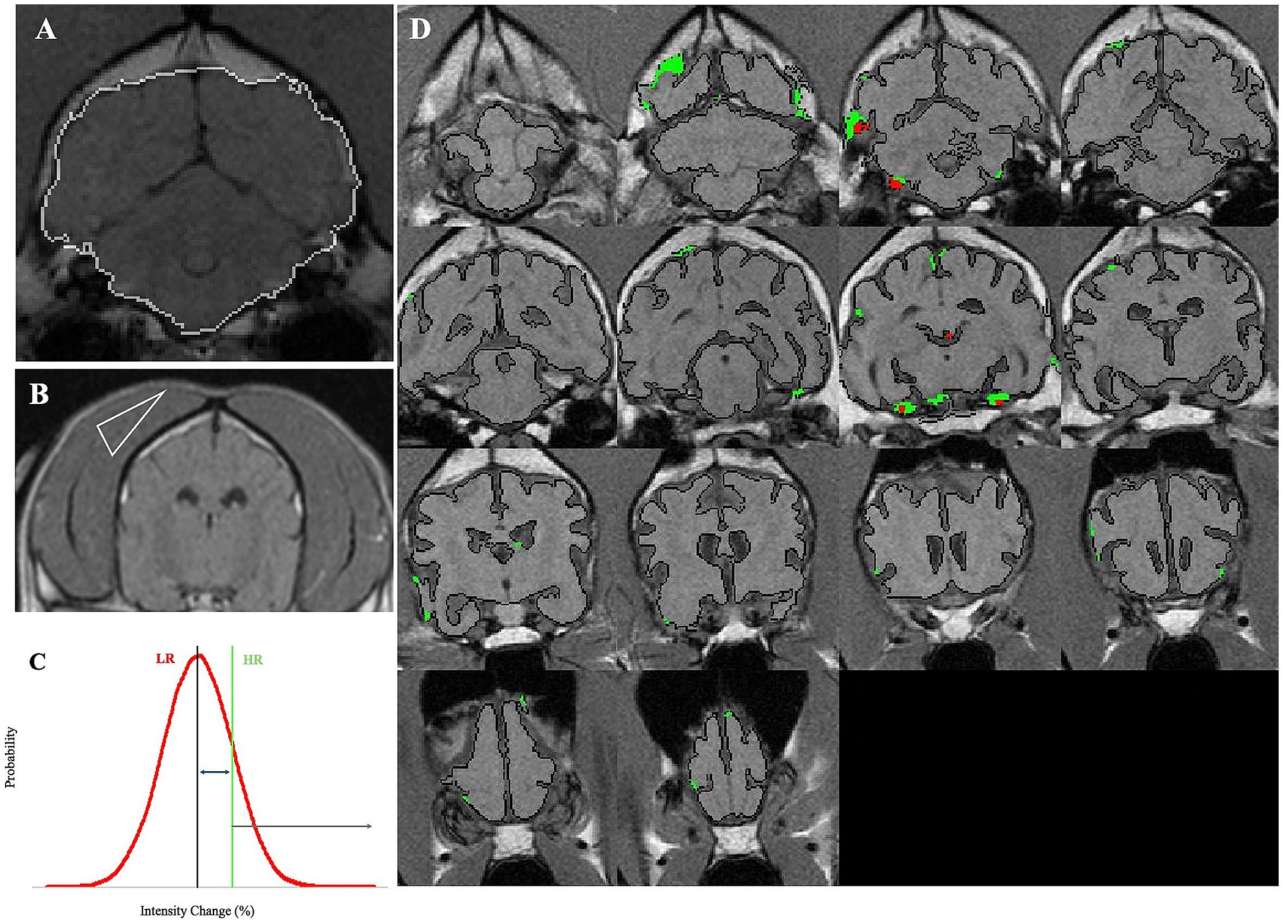
Demonstration of the analysis process of SEA. **(A)** White border line represents the masking of a brain slice, demarcated by the algorithm, and manually corrected when required. **(B)** A triangle-shaped slice from the temporal muscle was marked and used to account for unrelated physiological variations between dogs, such as blood pressure and perfusion rate. **(C)** High range (HR) was defined for values above the mean value of the temporal muscle plus one standard deviation. **(D)** LR-positive and HR-positive voxels were marked in green and red, respectively, to allow a detailed observation of areas with possible BBBD.

For each subject, transverse T1W images were analyzed using the following steps by a multistep in-house algorithm that was applied on brain slices (Figure 1):

1. Brain masking was performed by outlining brain tissue and was corrected manually to include only the brain tissue and exclude other surrounding tissues. The manual correction was conducted blindly on all scans.
2. For SEA, signal intensity differences were obtained based on each voxel’s pre-contrast intensity subtracted from the post-contrast intensity. The change in intensity percentage for each voxel was recorded. The equation used for calculation: [(post−pre)/pre]×100] . The change was expressed by means of the brightness of each voxel, indicating the accumulation of the contrast agent in the brain. At the end of this stage, each voxel received a value.
3. The temporal muscle of each dog was used as a reference value, because it represents a tissue with no blood–tissue barrier and is subjected to the unique physiological variables of each dog. A triangle-shaped slice from the temporal muscle of each dog was marked and used to account for unrelated physiological variations between dogs, such as blood pressure and perfusion rate (Figure 1B). A similar equation was used to calculate the differences in intensity percentage for each voxel of the temporal muscle. The mean and standard deviation of these values was calculated. Hence, a mean score of the temporal muscle and a standard deviation were determined for each dog based on the two thresholds that were set.
4. Two thresholds for permeable “positive” voxels were determined for each dog using the values calculated from the temporal muscle. Low range (LR) was termed the range between the mean intensity change (%) of temporal muscle voxels and the mean plus one standard deviation. This value is considered more sensitive, whereas the high range (HR) was defined for values above the mean value of the temporal muscle plus one standard deviation (Figure 1C). One (between the mean temporal muscle score and the mean +1 sd), and the value HR is considered less sensitive but may be more specific.
5. The percentage of all positive voxels from the entire brain was termed “BBB score.” Hence, each dog had two BBB score values: the HR BBB score and the LR BBB score.
6. Color-coded maps were produced. LR-positive and HR-positive voxels were marked in green and red, respectively, to allow a detailed observation of areas with possible BBBD (Figure 1D).
7. The same was performed for dogs in the control group, where a normal permeability value, calculated by similarly sampling the temporal muscle, based on which BBB scores were calculated for each control dog separately. The control group mean BBB scores plus two standard deviations were calculated and used as a threshold for normal BBB. Dogs in the study group with a mean value higher than the mean calculated for the control were considered to have BBBD.

### 2.4 Interthalamic adhesion diameter measurement

**Figure 2:**
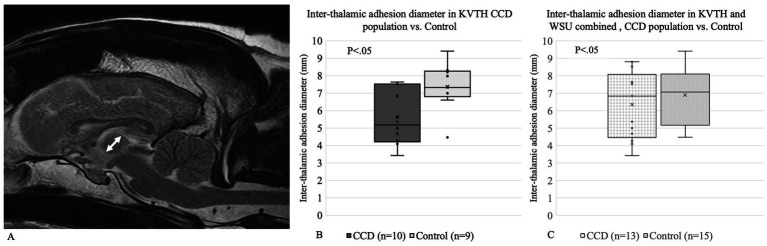
**(A)** Interthalamic adhesion diameter measurement for **(B)** Koret Veterinary Teaching Hospital and **(C)** Koret Veterinary Teaching Hospital and Washington State University combined.

Interthalamic adhesion measurements have previously been looked at as a potential biomarker for dogs with brain atrophy and potential associations with CCD; hence, we measured the diameter of interthalamic adhesion. The diameter of the interthalamic adhesion on T2-weighted images on the sagittal plane was measured on RADI-ANT DICOM © viewer software. Three measurements for each dog were performed blindly, and the average was calculated (Figure 2).

### 2.5 Statistical analysis

Statistical analysis was performed using RStudio (15). All statistical tests were performed with the Wilcoxon signed-rank test. The figures were generated using Excel (Microsoft 365, Version 16.87, NY, USA). Tests were performed separately for HR, LR, and interthalamic adhesion diameter between the respective sub-groups.

Magnetic resonance imaging data were analyzed by an author blinded to the group affiliation of the dogs. A *p*-value of 0.05 or less was considered statistically significant.

## 3 Results

### 3.1 Animals

A total of 28 dogs were included, with 19 dogs from the Koret Veterinary Teaching Hospital and 9 from Washington State University (WSU). Among these, 13 were included in the CCD group and 15 in the control group.

#### 3.1.1 KVTH

The CCD group included three male and seven female dogs. The dogs’ age ranged from 9 to 16 years (median age of 12 years). Eight dogs were mixed breeds, one was a terrier, and one did not have its breed reported. Body weight ranged between 6 and 35 kg (median 11 kg).

Of the control group, eight were male dogs, and one was a female dog. Four dogs were castrated, while five were intact. The dogs’ age ranged between 5 and 16 years (median 9 years). Three dogs were mixed breeds, and the remaining dogs were one of each of the following breeds: Bull Terrier, Labrador Retriever, Bernese Mountain dog, Cocker Spaniel, Maltese, and Vizsla. The body weight of these dogs ranged from 2 to 42 kg (median weight of 23 kg).

#### 3.1.2 WSU

The CCD group included three male dogs. Of these, two were castrated, while the status of the third was unknown. The ages of the dogs were 5, 9, and 11 years, and their breeds included Labrador Retriever, Vizsla, and Akita. The weight of these dogs was not provided.

The control group consisted of four female and two male dogs, all of which were spayed or neutered. Their ages ranged between 10 and 14 years (median age was 11.5 years). The breeds included one of the following: Affenpinscher, Shih Tzu, Border Collie, Terrier, a mixed-breed dog, and one that was of unknown breed. The weight of these dogs was not provided. Supplementary Table 1 depicts the different groups of dogs and clinical findings.

### 3.2 BBBD threshold measurements

(1) A high range (HR) score, considered a restrictive score, and (2) a low range (LR) score, considered a permissive score, was as follows: Koret-Control HR threshold—0.311. Koret-Control LR threshold—1.578.

To account for the age difference between dogs in the KVTH Control group being younger and the CCD group, median age of 9 years and 12 years, respectively, we created a sub-group of control dogs from KVTH that were 9 years old and older only. The thresholds score of KVTH-1 BBB for the older control threshold was HR—0.324, LR—1.679. Similarly, the average BBB scores of the WSU control group were also calculated and yielded the following thresholds: WSU—control HR was 2.851 and control LR was 2.443.

### 3.3 Dogs with indication of BBBD

Two of 10 dogs in the KVTH-CCD group had BBBD above the HR. The dog ages were 12 and 10 years old. Inter-thalmic adhesion diameters were 5.373 and 4.09 mm, respectively, shorter in diameter compared to the control group average.

Interestingly, of the three dogs in the WSU-CCD group, none were considered to show BBBD.

### 3.4 Comparison of BBB scores

**Figure 3:**
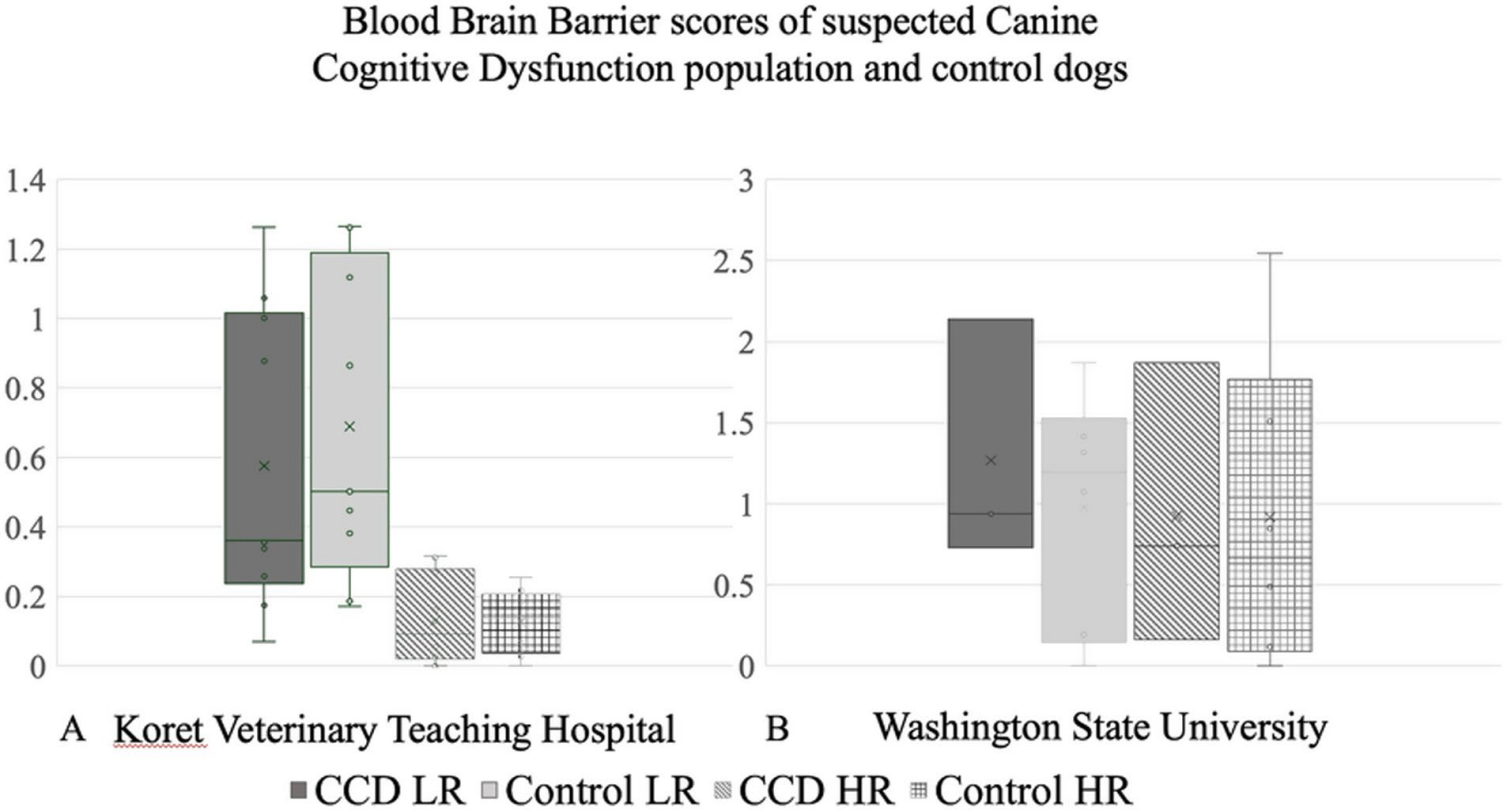
Blood–brain barrier scores comparing canine cognitive dysfunction population with control dogs in **(A)** Koret Veterinary Teaching Hospital and **(B)** Washington State University.

No significant differences were found between the mean BBB score value of the CCD dogs compared to the control group for both HR and LR in both institutions (Figures 3A,B).

Even when considering the age-matched control subgroup, no significant difference between the BBB scores of the CCD and control groups was noted.

### 3.5 Interthalamic adhesion

Dogs with CCD have been shown to have a smaller interthalamic adhesion diameter, which is considered a non-specific landmark for neurodegenerative processes. For KVTH, a significant difference was found between the interthalamic adhesion diameter of dogs with CCD (5.64 mm) compared to the control group (7.38 mm) (Figure 2A). Due to the low number of CCD dogs in WSU, no comparisons were performed as a separate group. When comparing the interthalamic adhesion diameter between KVTH CCD dogs (5.64 mm) and the KVTH subgroup of older controls (6.69 mm), statistical significance was lost. When combining the data from Koret and WSU, there were no significant differences between the groups (Figure 2B).

## 4 Discussion

In this study, we used an inhouse algorithm on a group of 28 dogs to examine the potential use of BBB permeability changes in MRI as a biomarker for CCD. We evaluated and quantified BBB permeability in dogs with CCD compared to dogs without CCD using SEA.

In our current study, BBB scores of dogs tentatively diagnosed with CCD were not significantly different than the normal dogs in the control group.

SEA, the main method of this study, has been applied similarly in previous studies and has been shown to increase the identification percentage of lesions in brain scans (12), as well as differentiate between brain gliomas and meningiomas (16).

Lack of differences between BBB scores of dogs with CCD and control, both for LR, which is more sensitive, and HR (considered more specific), could have arisen from several reasons. The BBB score was not calculated for separate brain regions in this study. In humans, BBB permeability was shown to be more prominent in men of a certain age compared to women, but these differences were only identified in the cingulate and occipital lobes and not in other brain areas (17). Another study found a higher BBB leakage rate in the cortex of Alzheimer’s disease patients compared to healthy individuals, but no difference was noted in the white matter (18). Future studies should look at specific brain areas related to CCD.

Using our SEA algorithm on this cohort of dogs did not detect a difference in BBB permeability when comparing young dogs (up to 8 years old) to old dogs (12 years and older), and did not find a correlation between age and BBB score. In contrast to our results, previous studies in humans have demonstrated that BBB permeability increases over time, resulting in detectable BBBD in older humans when compared to young adults (19, 20). Furthermore, a study performed on rats demonstrated that both AD and non-AD rats had an increase in the water BBB permeability, but it was found to occur earlier in AD rats (21). Thus, a compromised BBB is considered a part of the normal processes of aging, which is accelerated in AD patients (22). The variability of the lifespan of different breeds could have played a role in our results. In dogs, cognitive disorders are suspected to be more common in small breeds, as their lifespan is longer (2). It was suggested that large breed dogs start to age as soon as the age of 6 years, while small breeds at 10 years (23). Studies in veterinary medicine reported that aging is noted at 8 years (4) for 11 years (2). Our CCD group had a cutoff of 9 years old or older, except for one large breed dog, 5 years old, who showed progressive behavioral changes for a period of 6 months, and no abnormal findings were noted in his brain. It could be that, despite recognizing behavioral changes, in an older population, the BBBD changes would have been more pronounced. Finally, magnets with higher field strength (>3 T) be more sensitive in the detection of subtle differences in BBBD that were not depicted by lower strength magnets, as it allowed an increased signal-to-noise ratio at higher field strengths, while leakage can be subtle (24).

Interthalamic adhesion atrophy (11, 25) has been described as an anatomical feature correlating with brain atrophy, not specifically with CCD. In our study, we found a smaller interthalamic adhesion diameter in the CCD group from Koret, relatively to the control group. This result corresponds with previous findings, further supporting that interthalamic adhesion diameter may serve as a suitable parameter for the evaluation of brain atrophy in dogs suffering from CCD (11, 26). A negative correlation between the interthalamic adhesion diameter and the age of the dogs was noted in this study; however, it did not detect a difference when matched-age dogs with and without CCD were examined. Interthalamic adhesion may indeed be a good marker for brain atrophy, sensitive enough to detect a reduction in brain volume in dogs with normal aging. When both institutions’ data were combined, we did not find a smaller interthalamic adhesion diameter between the CCD compared to the control group. It may be that combining the information from two different imaging facilities with different magnet strengths, and hence different resolutions, decreases the ability to measure with the same level of precision. In addition, a bigger sample size would potentially limit this discrepancy.

Applying SEA on brain scans from both 0.35 T and 1.5 T MRI machines detected changes in the permeability of the BBB that could not be correlated to the clinical manifestation of CCD, age, or interthalamic adhesion diameter. Only two dogs diagnosed with CCD were classified as dogs with BBBD when the BBB score was calculated using the high range intensity threshold. This may indicate that there is a subgroup of dogs with CCD that demonstrates BBB leakage, which may or may not play a role in the pathological mechanism leading to the clinical syndrome.

## 5 Study limitations

Due to the retrospective nature of this study and ethical considerations, our control group did not include healthy dogs, but rather dogs that had head/brain MRI scans for clinical indications and were admitted for reasons not involving cognitive dysfunction, and where no brain pathology could be detected by the radiologist. Since the control dogs were chosen based on an MRI report and not having a presenting complaint related to behavioral changes, none of the control dogs were explicitly assessed for CCD and we cannot rule out that some of the elderly dogs did show mild signs of CCD not picked up by the owners and hence and might have contributed to the lack of significant differences.

Additionally, some dogs showing signs at ages earlier than expected for CCD, with the most significant dog presenting signs at 5 years of age, had normal MRI and cerebrospinal fluid; however, we cannot rule out the possibility of some dogs suffering from undiagnosed meningoencephalitis or other microscopic abnormalities without a brain biopsy or necropsy.

Combining data of images from two different MRI machines with different magnet strengths (i.e., 0.35 T and 1.5 T) is difficult and therefore should be taken with caution. Additionally, the different magnet strengths may have also contributed to the variations in measurements and the lack of significant findings.

The different BBB scores calculated from our two different institutions suggest we cannot determine a normal threshold of BBB scores for other studies to use, and that each study should calculate its normal threshold score.

SEA is a retrospective method analyzing BBB permeability, based on one post-contrast time point only. It could be that a larger number of dogs in both the control group and the CCD group would have allowed to determine a more accurate value for better characterization of BBB function in dogs with CCD. We could also contemplate based on this study, that BBBD in CCD are too subtle to be detected using SEA and a different technique such as the dynamic contrast-enhanced MRI could be more sensitive to depict the changes in the CCD brain and hence looking for static lesions/dysfunctions will have to change to dynamic studies even for diagnostic purposes.

## 6 Conclusion

In the current study, using SEA failed to demonstrate a significant increase in BBB permeability in our small cohort of dogs with CCD compared to dogs that served as controls. Nevertheless, two of the CCD dogs did demonstrate BBBD based on our threshold. Many studies support the use of CCD as a model for AD in humans. Dogs with CCD share many characteristics with human AD and may reveal innovations and therapeutic breakthroughs in people. Therefore, models of rodents and laboratory dogs are recently being replaced by models of pet dogs, which represent the pathophysiology of AD in a naturally occurring disease (2, 5). Knowing that the disrupted BBB plays a role in the pathophysiology of many neurodegenerative diseases (19, 20), there is a great need to advance BBB imaging methods for neurodegenerative disorders in dogs as well. This study was a preliminary attempt to check whether SEA is sensitive enough to detect changes in the BBBD in relation to CCD. Future studies should aim to validate these results using SEA on a larger group of dogs and compare to dynamic contrast imaging analysis (DCE-MRI) as a potentially more sensitive technique for the detection and characterization of BBBD in CCD.

## Data Availability

The original contributions presented in the study are included in the article/Supplementary material, further inquiries can be directed to the corresponding author.
