# Supplementary material for: Blood–brain barrier permeability changes in dogs with suspected canine cognitive dysfunction using magnetic resonance imaging subtraction enhancement analysis
Source: Front Vet Sci. 2025 May 13;12:1572286. doi: 10.3389/fvets.2025.1572286 (PMC12106434; doi:10.3389/fvets.2025.1572286)
Supplement: Supplementary file 1 [file Table_1.docx]

| **group** | **Institution** | **Age (Y)** | **Breed** | **Weight (Kg)** | **Clinical presentation** | **HR** | **LR** | **Inter-thalamic adhesion diameter (mm)** |
| --- | --- | --- | --- | --- | --- | --- | --- | --- |
| Study | 1 | 12 | MBD | 10 | Compulsive behavior | 0.0547 | 0.1734 | 7.56 |
| Study | 1 | 10 | NA | 8 | Restless, Circling | 0.3174 | 1.2631 | 4.09 |
| Study | 1 | 12 | MBD | 8 | Behavioral Changes | 0 | 0.3673 | 4.683 |
| Study | 1 | 16 | MBD | 14 | Behavioral Changes | 0.0381 | 0.2571 | 5.003 |
| Study | 1 | 9 | MBD | 23 | Behavioral Changes | 0.0258 | 0.3553 | 7.647 |
| Study | 1 | 14 | Terrier | 7 | Inadequate mentation | 0 | 0.0698 | 4.25 |
| Study | 2 | 11 | Labrador Retriever | - | Mentation changes | 1.8714 | 2.1396 | 8.793 |
| Study | 2 | 5 | Visla | - | Behavioral changes | 0.7399 | 0.9372 | 8.807 |
| Study | 2 | 9 | Akita | - | Dullness, incoordination | 0.1621 | 0.7307 | 8.527 |
| Study | 1 | 14 | MBD | 35 | Behavioral Changes | 0.2676 | 1.0582 | 7.523 |
| Study | 1 | 12 | MBD | 15 | Behavioral Changes | 0.312 | 1.002 | 5.373 |
| Study | 1 | 16 | MBD | 6 | Decreased mentation | 0.1289 | 0.8774 | 3.43 |
| Study | 1 | 12 | MBD | 12 | Behavioral Changes | 0.1603 | 0.3375 | 6.84 |
| Control | 1 | 16 | MBD | 23 | ODVS | 0.0453 | 0.502 | 6.617 |
| Control | 1 | 16 | Bull terrier | 16 | ODVS | 0.2555 | 1.118 | 7.003 |
| Control | 1 | 8 | Labrador Retriever | 41 | ODVS | 0.1961 | 0.4475 | 7.987 |
| Control | 1 | 5 | Bernese Mountain Dog | 36 | Limb weakness | 0.1379 | 1.2627 | 8.207 |
| Control | 1 | 9 | Cocker spaniel | 23 | ODVS | 0.0266 | 0.1865 | 4.473 |
| Control | 1 | 5 | Maltese | 2 | Limb weakness | 0.067258 | 0.382146 | 7.337 |
| Control | 2 | 14 | Affenpinscher | - | Vestibular signs | 0 | 0 | 5.173 |
| Control | 1 | 10 | Visla | 42 | Blindness | 0.1829 | 1.2652 | 8.31 |
| Control | 1 | 13 | MBD | 14 | ODVS | 0 | 0.1717 | 7.063 |
| Control | 1 | 6 | MBD | 35 | Otitis media | 0.217 | 0.8647 | 9.407 |
| Control | 2 | 11 | Shih Tzu | - | Limb weakness | 0.8482 | 1.073 | 4.96 |
| Control | 2 | 10 | Border Collie | - | excessive panting | 0.1183 | 0.1916 | 8.7 |
| Control | 2 | 12 | NA | - | Collapsing in hindlegs | 0.4876 | 1.3173 | 8.107 |
| Control | 2 | 11 | MBD | - | Hindlimbs weakness | 2.5448 | 1.8689 | 4.89 |
| Control | 2 | 12 | Terrier | - | Acute vestibular signs, IVDD | 1.5102 | 1.4154 | 6.247 |

Supplementary Table 1: Data collection.

Institution: 1, Koret Veterinary Teaching Hospital, 2. WSU Veterinary Teaching Hospital, MBD, Mix Breed Dog, NA, Not Applicable, ODVS, suspected Old Dog Vestibular Syndrome
